# Improving the prediction of the functional impact of cancer mutations by baseline tolerance transformation

**DOI:** 10.1186/gm390

**Published:** 2012-11-26

**Authors:** Abel Gonzalez-Perez, Jordi Deu-Pons, Nuria Lopez-Bigas

**Affiliations:** 1 Research Programme on Biomedical Informatics - GRIB. Universitat Pompeu Fabra - UPF, Hospital del Mar Medical Research Institute - IMIM. Parc de Recerca Biomèdica de Barcelona (PRBB). Dr. Aiguader, 88, E-08003 Barcelona, Spain; 2 Institució Catalana de Recerca i Estudis Avançats (ICREA). Passeig Lluís Companys, 23, E-08010, Barcelona, Spain

## Abstract

High-throughput prioritization of cancer-causing mutations (drivers) is a key challenge of cancer genome projects, due to the number of somatic variants detected in tumors. One important step in this task is to assess the functional impact of tumor somatic mutations. A number of computational methods have been employed for that purpose, although most were originally developed to distinguish disease-related nonsynonymous single nucleotide variants (nsSNVs) from polymorphisms. Our new method, transformed Functional Impact score for Cancer (transFIC), improves the assessment of the functional impact of tumor nsSNVs by taking into account the baseline tolerance of genes to functional variants.

## Background

With the advent of high-throughput sequencing, our ability to identify single nucleotide variants (SNVs) in the genome or exome of individuals has far exceeded our capacity to experimentally validate their impact on disease phenotypes. Therefore, computational methods that predict the impact of non-synonymous SNVs (nsSNVs) on protein function have become very important and of wide interest. Bioinformatics methods have been developed and tested over the past decade that distinguish disease-related nsSNVs from neutral polymorphisms [1-11]. A different, although related, problem is assessing the relevance of nonsynonymous somatic variants in cancer emergence. In principle, functional somatic mutations can only be causative of cancer if they affect cancer driver genes, which upon mutation confer a distinct selective advantage or a newly acquired capability to the cell [12,13].

The need for computational methods to predict the functional impact of cancer-causing somatic variants contrasts with the low number of methods that have been designed or tested specifically for this purpose [4,14]. One likely explanation is the absence of curated sets of true driver and passenger cancer mutations. Many recently published cancer resequencing projects use methods like SIFT [15,16], and PolyPhen2 (PPH2) [17-19] to predict the functional impact of cancer somatic mutations, although these methods were not developed or tested for this purpose and the quality of their performance in this context is not clear.

Existing methods provide a predictive functional impact score (FIS) for each mutation [3]. The FIS calculated for nsSNVs relies mainly on the conservation of single residues across multiple sequence alignments. In other words, these methods employ evolutionary information to assess the likely impact of an amino acid change on the structure or function of the altered protein. Nevertheless, the ultimate effect of this amino acid change on the functioning of a cell depends on other factors as well, such as the particular role played by the altered protein in the cellular machinery. The criticality of that role will determine the protein's tolerance to amino acid changes. Our view is that a score purporting to assess the likelihood of individual mutations to provide a somatic cell with an acquired advantage - and possibly give origin to a tumoral clone - must take this feature into consideration.

The present study has two interrelated goals: first, to determine the tolerance of different proteins to functional variants, and second, using this information to develop a method that improves the capacity of existing bioinformatics tools to assess the likelihood that a specific somatic mutation is a cancer driver. We have called it transFIC (transformed Functional Impact Scores in Cancer) and we distribute it as a PERL script that users can download for local use. We also provide a web server [20] that can be queried using an internet browser or programmatically to obtain the transFIC of somatic cancer nsSNVs.

## Materials and methods

### Obtaining and processing nsSNVs from 1000 Genomes

We downloaded all SNVs (approximately 30 million) detected by the 1000 Genomes Project [21] within the genomic sequences of 1,197 individuals (May 2011 release). We then used the Ensembl Variant Effect Predictor [22,23] (VEP v.62) to detect nsSNVs and to retrieve their SIFT [1,24] and PPH2 [2] FISs. We retrieved the corresponding MutationAssessor (MA) FISs through the MA webAPI service (release 1.0) [3]. At the end of this process we obtained 168,803 distinct SNVs, of which 155,453 were successfully scored by at least one method and 110,397 were scored by all three methods.

### Computing the FIS distribution of groups of functionally related genes

**Figure 1:**
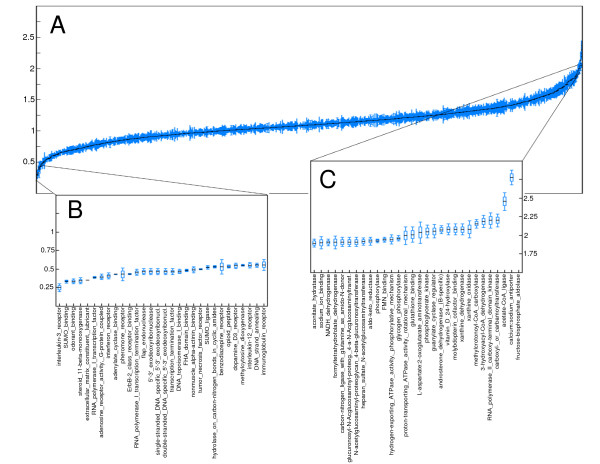
**The distribution of MutationAssessor functional impact scores of nonsynonymous single nucleotide variants differs significantly in proteins belonging to different functional groups**. **(a) **Candlestick representation of the distributions of MutationAssessor (MA) scores of germline single nucleotide variants (SNVs) in genes in all Gene Ontology Molecular Function (GOMF) categories, ordered from higher to lower mean. **(b,c) **Thirty least-tolerant and 30 most-tolerant GOMF groups of nsSNVs ordered by their mean MA scores. Groups in the lower end of the tolerance scale (less tolerant) correspond to essential GOMF categories, involved in signal transduction, transcription, and translation. On the other hand, the most tolerant molecular functions correspond mainly to metabolic-related activities.

We obtained Gene Ontology Biological Process (GOBP) and Molecular Function (GOMF) categories [25], canonical pathways (CP) [26] and Pfam domain (Dom) [27] annotations for all protein-coding genes included in Ensembl v.62 from the Ensembl Biomart service [28], MsigDB (a database that maintains several collections of gene signatures) [26] and the Pfam database (which included the information on domain borders) [27]. Finally, we grouped together the nsSNVs that occur in the genes assigned to each category of these four annotation systems. (The distribution of FISs of the nsSNVs in different functional categories are shown as candlesticks in Figure 1 and Additional files 1 to 3.)

We then built one FIS distribution for each human protein-coding gene following this simple pipeline (see the section 'Using baseline tolerance to functional variants to transform original scores' in the Results and discussion for an example).

#### Step 1

We obtained all the functional terms assigned to the gene under analysis by each of the four functional annotation systems. If an annotation system contained no annotation for a particular gene, the pipeline was stopped at this stage and the mean and standard deviation of FISs of the germline nsSNVs tolerated by the gene were taken from the values corresponding to the distribution of the entire dataset of nsSNVs.

#### Step 2

From the list retrieved for the gene of interest in step 1, we culled the SNVs that occur in genes annotated to the most specific functional term (that is, the term containing the fewest genes).

#### Step 3

If we culled fewer than 20 SNVs, step 2 was repeated including the SNVs occurring in the genes annotated to the second functional term in ascending order of gene content, then SNVs of genes in the third category with the fewest genes, reiterating the process until at least 20 nsSNVs scored by the three methods were pooled. The aim was to obtain for each gene a pool of nsSNVs large enough to compute the FIS distribution, but as small as possible to permit a better grasp of the gene's baseline tolerance of mutations related to its specific function.

#### Step 4

The SIFT and PPH2 FISs underwent a logit transformation to approximate them to a normal distribution.

#### Step 5

The mean and standard deviation of the FISs provided by each method were calculated within the pooled set.

This process yielded four output files, each containing the mean and standard deviation of the three FIS distributions (one for each method) assigned to each human protein-coding gene that completed this pipeline. These values can be easily used to transform the scores of somatic mutations as described in the equation in the Results and discussion section. The PERL script simply reads the SIFT, PPH2 and MA FISs that assess the functional impact of the somatic mutation under analysis, searches the distributions that have been assigned to the gene where that somatic mutation occurs and automatically transforms the original FIS.

### Assembling proxy datasets to test the performance of transFIC

From version 57b of the Catalog of Somatic Mutations in Cancer (COSMIC), we downloaded all somatic nSNVs. We then counted the number of samples containing each mutation. We obtained SIFT and PPH2 FISs from the Ensembl VEP v.62 and MA FISs by querying the MA webAPI, as described above.

We assembled the whole genome (WG) dataset by downloading the coordinates of somatic mutations from the International Cancer Genome Consortium (ICGC) Data Coordination Center [29] or from the data provided with the software implementing the MEMo algorithm [30] (Table 1) of 12 cancer exome (or selected genes) sequencing projects. (The MEMo algorithm is designed to find highly interconnected mutually exclusive cancer driver genes.) Mutations in hg18 coordinates were transformed to hg19 using the Liftover program obtained from the UCSC genome browser [31]. The SIFT, PPH2 and MA FISs were then obtained as described above.

**Table 1:** Number of somatic mutations contributed by 12 cancer genome-sequencing projects to conform some of the proxy datasets

Tumor datasets	Samples analyzed	Genes with non-synonymous mutations	Non-synonymous mutations	Center	Source
breast(JHU)	39	483	649	Johns Hopkins University	ICGC DCC
breast(WTSI)	100	3644	5,189	Sanger Center (ICGC)	ICGC DCC
ovary(TCGA)	316	7082	12,819	TCGA	MEMo
CLL(MICINN)	109	944	1,160	MICINN (ICGC)	ICGC DCC
colorectal(JHU)	34	415	600	Johns Hopkins University	ICGC DCC
pediatricbrain(DKFZ)	109	604	730	DKFZ (ICGC)	ICGC DCC
glioblastoma(TCGA)	139	400	740	TCGA	MEMo
glioblastoma(JHU)	77	1,269	1,536	Johns Hopkins University	ICGC DCC
lung(TSP)	153	320	755	Washington University School of Medicine	ICGC DCC
pancreatic(JHU)	112	737	962	Johns Hopkins University	ICGC DCC
pancreatic(OICR)	34	1,361	1,792	OICR (ICGC)	ICGC DCC
pancreatic(QCMG)	67	847	1,033	QCMG (ICGC)	ICGC DCC

Only mutations successfully scored by at least one method were included. Original sources: breast(JHU) [40,41], breast(WTSI) [42], ovary(The Cancer Gene Atlas) [43], CLL(MICINN) [44,45], colorectal(JHU) [40,46], pediatricbrain(DKFZ) [47,48], glioblastoma(TCGA) [49], glioblastoma(JHU) [50], lung(TSP) [51], pancreatic(JHU) [52];, pancreatic(OICR) and pancreatic(QCMG) are unpublished lists of mutations downloaded through the ICGC data coordination centre [29]. DKFZ, German Cancer Res Center; ICGC, Data Coordination Center [29]; MEMo, datasets of mutations packed with the software implementing the MEMo algorithm [30]; MICINN, Spanish Ministry of Science and Innovation; OICR, Ontario Institute for Cancer Research; QCMG, Queensland Centre for Medical Genomics.

We obtained a list of driver cancer genes from the Cancer Gene Census (CGC) [13]. Somatic mutations from COSMIC and from the WG dataset that appeared in any of the genes in the CGC constituted the positive subsets of two proxy datasets. The negative subsets were composed of COSMIC or WG somatic mutations occurring in other genes and are not recurrent in the corresponding dataset (Table 2).

**Table 2:** Composition of the datasets used as proxies to compare the performance of transformed and original scores at assessing the functional impact of cancer somatic mutations

Name	Source	Positives	Negatives	N positives	N negatives
COSMIC2+/1	COSMIC	Mutations that appear in 2 or more samples	Mutations that appear in 1 sample	4,012	39,854
COSMIC5+/1	COSMIC	Mutations that appear in 5 or more samples	Mutations that appear in 1 sample	1,480	39,854
COSMIC2+/Pol	COSMIC/ HumVar [2]	Mutations that appear in 2 or more samples	Known polymorphisms	4,012	8,257
COSMIC5+/Pol	COSMIC/ HumVar	Mutations that appear in 5 or more samples	Known polymorphisms	1,480	8,257
COSMICD/O	COSMIC	COSMIC mutations included in the manually curated list of drivers used to train CHASM [5]	COSMIC mutations without the positive subset	2,185	41,681
COSMICD/Pol	COSMIC/ HumVar	Mutations included in the manually curated list of drivers used to train CHASM	Known polymorphisms	2,185	8,257
COSMICCGC/ nonCGC	COSMIC	COSMIC mutations in genes included in the Cancer Gene Census [13]	Non-recurrent COSMIC mutations in genes not included in the Cancer Gene Census	4,685	35,907
WG2+/1	Pooled cancer somatic mutations	Mutations that appear in 2 or more samples	Mutations that appear in 1 sample	1,031	26,025
WGCGC/ nonCGC	Pooled cancer somatic mutations	Mutations in genes included in the Cancer Gene Census [13]	Non-recurrent mutations in genes not included in the Cancer Gene Census	1,412	24,837

HumVar is a dataset of disease-related SNVs and neutral polymorphisms [2]. WG (whole genome) is a dataset of somatic mutations pooled from different tumor exome-sequencing projects (Table 1).

In summary, recurrent COSMIC or WG mutations, manually curated driver mutations within COSMIC or mutations within COSMIC or WG occurring in CGC genes formed the positive subsets of the nine proxy datasets employed (Table 2), whereas the negative subsets were formed by non-recurrent COSMIC or WG mutations, COSMIC mutations outside the manually curated drivers list, non-recurrent COSMIC or WG mutations in non-CGC genes, or known polymorphisms.

Finally, we downloaded and ran the CHASM program [5,14,32]. Following the recommendation by the developers in their wiki pages, we used the ovarian classifier to classify mutations within these two datasets. Next we computed the transFIC (GOMF) of CHASM using the procedure described above. To evaluate the performance of the original and transFIC score we used WG2+/1 and WGCGC/nonCGC datasets, and a modification of them in which we removed the mutations that appear within the training set of CHASM (WG2+/1* and WGCGC/nonCGC*). Manually curated driver mutations used to train CHASM were identified within COSMIC to serve as the positive subset in two of the proxy datasets (COSMICD/O and COSMICD/Pol).

## Results and discussion

### Hypothesis

We hypothesized that we could use the pool of nsSNVs that occur naturally in human populations to assess gene tolerance to perturbing nsSNVs. Since in principle all nsSNVs that interfere with the natural development of a human organism or with its ability to produce offspring have been eliminated from this pool by negative selection, the range of nsSNVs that remain in a gene would reflect the 'baseline tolerance' of the cell or organism to perturbations to the function of that gene. We propose that this baseline tolerance can complement the evaluation of violations of evolutionary constraints imposed on individual amino acid residues by protein structure and function. Therefore, we propose to use it as a means to transform the FISs of nsSNVs provided by bioinformatics tools.

One way to visualize the score transformation that we propose is that mutations with the same FIS should affect the cell differently if they occur in genes encoding essential proteins rather than in genes with numerous backup and redundancy mechanisms - for instance, those with a higher degree of paralogy. Our assumption is that genes within the former class will mainly possess germline SNVs with relatively low FISs, while those within the latter will accumulate more functional SNVs. To accomplish this transformation we devised two interrelated objectives: first, to measure whether this baseline tolerance to nsSNVs actually differs for distinct genes, and second, to evaluate whether a differential baseline tolerance to SNVs could be used to improve the scoring of functional somatic mutations in cancer. To carry out the study, we selected the nsSNV FISs provided by SIFT [1,24], PPH2 [2] and MA [3] because they can be readily obtained for high-throughput analysis of large datasets of mutations, a critical feature for somatic mutation analysis in the context of cancer genome resequencing projects.

Although cancer-related genes are better conserved than average human genes [33,34], which has aided in the discovery of new cancer genes [35], to our best knowledge this is the first attempt to evaluate whether baseline tolerance to germline SNVs can improve the FIS of somatic mutations.

### Detecting differences in baseline tolerance across genes

To detect differences in baseline tolerance across genes, we first needed a pool of nsSNVs that occur naturally across human populations. We decided to use the catalog of SNVs detected by the 1000 Genomes Project [21] because of its unbiased nature. However, the number of nsSNVs deposited in this catalog does not allow computation of each individual gene's baseline tolerance, because the catalog still lacks the necessary coverage. Therefore, we clustered the genes according to functional criteria (as described in Materials and methods) and then computed the baseline tolerance of these groups of functionally related genes. This approach must be seen only as an imperfect effort to compensate for the low resolution of our current catalogs of SNVs, which prevents gene-by-gene calculation of baseline tolerance to SNVs. Nevertheless, as the genomes of more individuals are sequenced and the catalog of human germline nsSNVs progresses toward completion, eventually this assessment will become possible.

The four systems of functional annotation we used to partition the dataset of SNVs and form these pools of functionally related genes were (as introduced in Materials and methods) the GOBP and GOMF categories, the CP annotations and Doms. Let us illustrate this process with the GOMF terms represented in Figure 1. Each of these terms contains a group of functionally related human protein-coding genes. The nsSNVs that occur in these genes are pooled together to build the distribution of the three FIS values (one for each bioinformatics tool assayed) in each category. Then, the distribution of, for instance, MA scores for the nsSNVs that occur in the genes of each GOMF group may be represented as a candlestick centered at the mean of the distribution, and whose whiskers extend outward in proportion to the standard error of the mean of the distribution. If the groups are ordered in ascension by their MA score means, we obtain the plot shown in Figure 1a. The group located at the extreme left of the graph (interleukin-3 receptor) possesses nsSNVs with lower MA scores, on average, than its counterpart at the extreme right of the graph (immunoglobulin receptor). Genes in GOMF groups at the extreme left of Figure 1a have lower tolerance to perturbing nsSNVs (they have, on average, lower mean MA scores) than those at the extreme right, which tend to bear more deleterious nsSNVs.

We have observed that this same segregation between genes with low baseline tolerance and genes with high baseline tolerance holds if the genes and the nsSNVs they bear are grouped following other functional classification systems (Additional files 1 to 3). For example, canonical pathways (Additional file 1) that group genes related to biological processes such as cell cycle, central signal transduction pathways, or DNA damage repair are located at the lower end of the MA score spectrum, which means that only germline SNVs with relatively low functional impact are tolerated in these genes. On the other hand, most metabolic pathways appear to tolerate germline SNVs with higher functional impact, as they are primarily located at the upper end of the MA score spectrum. This finding may be related with the fact that many known inherited metabolic disorders are known to be recessive [36] (see below).

The distributions of MA scores of nsSNVs across all GOBP and Dom groups, which follow this same general structure, are presented in Additional files 2 and 3. A comparison of the MA baseline tolerance of genes - the mean MA score of SNVs - assigned according to the GOBP and the GOMF pooling (Additional file 4) shows some weak correlation between the two. Even weaker correlations are observed when the other classification schemes are compared to the baseline tolerance according to GOBP. These differences in baseline tolerance measurements are probably the reason why dissimilar classification schemes perform differently when separating the proxy datasets (see below).

In an effort to understand the reasons for these observed differences in baseline tolerance between diverse groups of genes and especially whether they could be the product of artifacts in the data, we analyzed their correlation with several variables. We found that differences in baseline tolerance between groups of proteins cannot be explained by differences in the height of multiple sequence alignments used to produce the MA scores. Baseline tolerance also does not correlate with nsSNVs or allelic frequency. However, genes in the least tolerant groups are significantly more conserved, on average, than genes of the most tolerant groups. This is in agreement with our hypothesis that genes with low baseline tolerance are more critical to the cell - and therefore tend to evolve at a slower rate - than those exhibiting high baseline tolerance to nsSNVs. On the other hand, we found that dominant disease genes are significantly overrepresented among least tolerant genes and recessive disease genes are overrepresented among the most tolerant genes. In addition, known cancer genes are overrepresented in the least tolerant groups with respect to most tolerant groups. However, tumor suppressor genes and oncogenes are not significantly enriched for amongst lowly tolerant or highly tolerant GOMF groups (Additional file 5).

### Using baseline tolerance to functional variants to transform original scores

We wanted to transform the FISs of SNVs provided by SIFT, PPH2 and MA by taking into account these differences in tolerance to functional mutations in the germline. We are using the generic term 'functional impact score' - originally employed by the MA team [3] - to refer to the scores provided by these various methods. The rationale behind the transformation is that if two mutations with the same FIS affect genes with different germline tolerance to functional SNVs, the impact of the mutation on the least tolerant gene is expected to be greater than its impact on the most tolerant one. If GOMF results are taken as reference (Figure 1), a mutation on a gene with one of the functions shown in Figure 1b is expected to have a higher impact than another mutation affecting a protein with a function shown in Figure 1c.

As explained above, another way to present this transformation is to think of it as adjusting the FIS of the mutation to compensate for the importance of the gene to cell operation. Genes with essential cellular functions would appear on the lower end of the functional impact score scale, while genes whose malfunction can be compensated for by diverse mechanisms or does not lead to very deleterious phenotypes are located at the upper end of the FIS scale.

**Figure 2:**
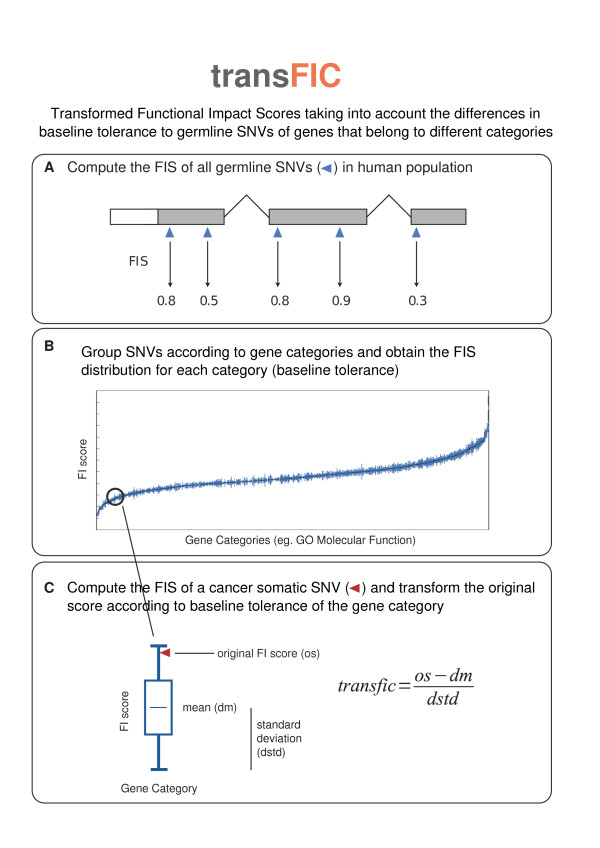
**Outline of the method to transform the scores**. **(a) **Functional impact scores (FISs) of all germline single nucleotide variants (SNVs) from the 1000 Genomes Project are computed. **(b) **SNVs are partitioned into subsets according to the category of the genes that harbor them (for example, Gene Ontology Molecular Function). **(c) **FISs of a given cancer somatic mutation are computed and transformed using the distribution of the scores of SNVs in the same category as the protein where the mutation under analysis occurs. We give these transformed scores the generic name transFIC (transformed Functional Impact scores in Cancer).

Figure 2 presents the flowchart used to transform the original FIS. Let us illustrate this process with one specific *PIK3CA *mutation detected in breast invasive carcinoma by the The Cancer Gene Atlas. This particular mutation involves the change of the glutamic acid residue at position 545 of the protein to an alanine residue. The MA FIS for this mutation is 1.775, which makes it a low impact mutation.

First, we compute the functional impact for all germline SNVs detected in the human population (1000 Genomes Project) using SIFT, PPH2 and MA (Figure 2a). Next, a measure of baseline tolerance to germline SNVs is computed for each protein-coding gene. We do this by pooling all genes with GOMF terms shared by the gene in question and computing the means and standard deviations of the FISs of the nsSNVs that affect them (as detailed in Materials and methods; Figure 2b). In this example, *PIK3CA *belongs to nine GOMF terms of increasing hierarchy from 'phosphatidylinositol-4,5-bisphosphate 3-kinase activity', which contains only five scored nsSNVs, to 'protein binding', with more than 9,500 scored nsSNVs. Pooling the scored nsSNVs of the three most specific GOMF terms (phosphatidylinositol-4,5-bisphosphate 3-kinase activity, 1-phosphatidylinositol-3-kinase activity, inositol or phosphatidylinositol kinase activity) satisfies the condition of using at least 20 nsSNVs to compute the baseline tolerance of a gene. In the case of *PIK3CA*, grouping these nsSNVs yields mean and standard deviation MA FISs of 0.853 and 0.327, respectively. (An analogous algorithm is applied to compute the baseline tolerance of genes in accordance to the three other classification systems.)

Finally, the original FIS of a cancer somatic SNV is transformed according to the baseline tolerance of the gene that harbors it, as illustrated by the following equation (Figure 2c):

$$transfic=\frac{os-dm}{dstd}$$

where *transfic *represents the transformed FIS, *os *the original score, and *dm *and *dstd *are the mean and the standard deviation of the distribution of FISs of nsSNVs computed as explained above. In our illustrative example, this implies that the E545A mutation in *PIK3CA *will see its MA FIS score of 1.775 converted to a transFIC MA of 2.82, which being above 2 will be labeled by the transFIC webserver as a highly affecting mutation (see below). In the spirit of our interpretation of the transFIC presented in the Hypothesis section, we may propose that, in this case, the transFIC compensates for the effect of a relatively mild mutation - one that occurs at a site of the gene does not possess strong evolutionary constraints. The resulting transFIC would thus more accurately present the impact of this relatively mild malfunction of a key signaling protein - whose essentiality is reflected in its low baseline tolerance - on cell operation.

### Comparing transformed FISs to original FISs

To compare the capability of the transformed FIS to that of the original FIS to identify mutations involved in cancer, we needed a set of somatic mutations involved in cancer development (positive set) and a set of passenger somatic variants (negative set); however, no gold-standard dataset exists. Previously used datasets are based on the recurrence of mutations found in the COSMIC database [3,37] or manually curated sets of cancer driver mutations [5,14]. However, each of these datasets has its own biases; in particular, they are enriched for mutations in well-known genes that have been widely studied in cancer. Instead of employing only one data source, we decided to use several proxy datasets with nsSNVs gathered from different sources, under the assumption that each will have its own biases and errors.

We devised these proxy datasets so that the positive subset of mutations is enriched in likely driver mutations - either because they have been manually curated from previous reports, because they occur in known cancer genes, or because they appear recurrently in the dataset - and is complemented by a negative subset of mutations enriched in passenger mutations. Known driver mutations are the result of years of cancer genetic and genomics research and are, in most cases, experimentally verified [5]. Mutations that occur in cancer genes have an increased likelihood of being drivers because they are prone to affect likely driver genes. On the other hand, mutations that recur in several different tumors also have an increased likelihood of being drivers, because their increased frequency makes it more likely that they have been positively selected and less likely to have appeared by chance in tumors. As stated above, both recurrent cancer mutations and mutations in cancer genes have been employed elsewhere as datasets enriched in driver mutations.

Some of these proxy datasets are derived from COSMIC version 57b [35], while others come from a pool of nonsynonymous somatic mutations detected by 12 whole-exome (or comprehensive specific gene) tumor sequencing projects framed within the ICGC [38] and The Cancer Gene Atlas. The number of nonsynonymous somatic mutations obtained from each cancer genome re-sequencing project included in the pool dataset are detailed in Table 1. The composition of all the proxy datasets is listed in Table 2 and their assembly is described in detail in the Materials and methods section. The negative subset in some datasets is composed of known polymorphisms [2]. Furthermore, we provide the subsets of mutations that compose these nine proxy datasets in the help section of the transFIC web page [20], because we consider they could be useful for other researchers interested in developing methods to identify cancer driver variants. (The names of the subsets respect the nomenclature from Table 2.)

By using several datasets derived from different sources we can assess if the transFIC works systematically better than the original FIS. In other words, we assume that each dataset has an unknown percentage of misclassified mutations. For this reason, instead of focusing on the net performance of each method in a particular dataset we look for the systematic improvement of the transformed FIS.

We computed the transformed FIS of all somatic mutations in the nine proxy datasets. To assess the performance of each FIS (or transformed FIS) in identifying likely functional somatic mutations, we computed the Matthews correlation coefficient (MCC) and overall accuracy (ACC) yielded by the classification of positive and negative cases in each proxy dataset. We did this calculation for cutoff values covering the full range of FIS (or transformed FIS) and retained the highest MCC achieved and the ACC corresponding to the same cutoff value. The MCC and ACC were computed using:

$$MCC=\frac{tp\times tn-fp\times fn}{\sqrt{\left(tp+fp\right)\left(tp+fn\right)\left(tn+fp\right)\left(tn+fn\right)}}$$

and

$$ACC=\frac{tp+tn}{tp+fp+fn+tn}$$

where *tp*, *tn*, *fp *and *fn *are the number of true positive, true negative, false positive and false negative cases detected by the FIS (or transformed FIS) in question. Because all datasets are relatively biased towards an excess of negative cases, the MCC is a better estimator of performance than accuracy [6].

**Figure 3:**
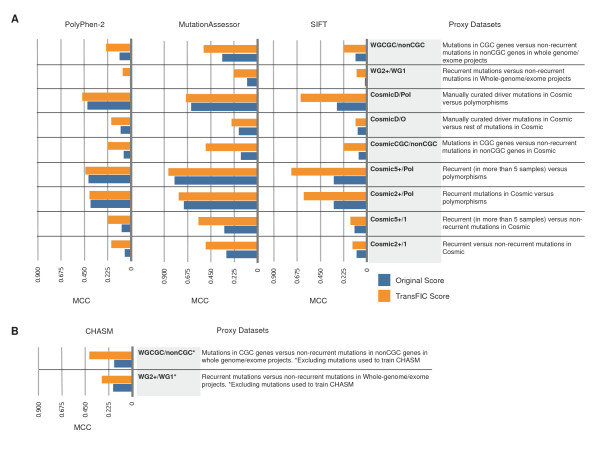
**Transformed Functional Impact for Cancer (transFIC) systematically outperforms original scores in the task of differentiating cancer driver mutations from neutral variants**. **(a) **Performance of GOMF transFIC is compared to the three original functional impact scores (FISs) classifying the nine proxy datasets, using as cutoff the value of FIS (or transFIC) that maximizes the Mathews correlation coefficient (MCC) in each case. **(b) **Performance of GOMF transFIC is compared to the original score of CHASM (q-value cutoff <0.05) in two proxy datasets after removal of mutations within the training set of CHASM.

We found that the transformed FIS outperforms the original FIS on all nine proxy validation sets. In the case of MA, this is true for transformed FISs computed from the GOMF partition for all proxy datasets tested (Figure 3; Additional file 6), while the gain is more modest or nonexistent when other partitions are used. In the case of PPH2 and SIFT the transformed FISs systematically outperform their original counterparts in all partitions and all proxy datasets tested, with up to 12-fold improvement in some cases.

Since PPH2 was trained using HumVar polymorphisms, we did an additional validation of this method using polymorphisms not present in the HumVar dataset (Additional file 6). The MCC of the original score of PPH2 diminished in these datasets but interestingly transFIC score performed comparably.

It is important to note that the transformation of the FIS affects the SNVs in all proxy datasets equally, depending solely on the functional annotation of the gene where the SNV is located. In other words, a somatic mutation (from COSMIC, for instance) and a common polymorphism (from HumVar) will see their FISs diminished in exactly the same quantity if they occur in genes within the same functional category. The resulting transFIC computed for the polymorphism in this hypothetical example will be probably lower than the transFIC of the cancer mutation, but only because the original FIS of the polymorphism was closer to the baseline tolerance of its gene.

Next we decided to test the transFIC approach with CHASM [5,14,32], a method trained to distinguish manually curated driver mutations from randomly generated mutations. Thus, in this case, the distribution of CHASM scores across GOMF groups computed from 1000 Genomes Project nsSNVs reflects the tolerance of different functional groups of genes to driver-like mutations in the human population. We used WG2+/1 and WGCGC/nonCGC datasets to test CHASM and the transFIC of CHASM. Note that since CHASM was trained with manually curated driver mutations from COSMIC we could not use COSMIC datasets to assess CHASM; moreover, the datasets used were modified to eliminate the mutations that appear within the training set of CHASM. The result of this assessment shows that the transFIC of CHASM outperforms significantly the original score in the two datasets tested (Figure 3b; Additional file 7). Thus, the transFIC approach is also useful to transform scores that prioritize driver-like nsSNVs.

One final remark that must be made about the transFIC approach is that it is not in principle specific to cancer somatic mutations. Although our basic interest, as stated in the Hypothesis section was to improve the FIS provided by known tools that rank cancer mutations according to their likelihood of being drivers, in principle the basic reasoning on baseline tolerance may be applied also to disease mutations as well. This is why we tested the performance of the transformed FIS of SIFT, PPH2 and MA on the classification of HumVar, a dataset of disease-related/neutral nsSNVs [2]. We found no improvement whatsoever with respect to their original counterparts (Additional file 8). To understand the reason for this result, we checked the distribution of disease-related genes (those annotated in OMIM [39]) across the GOMF groups ordered by baseline tolerance, as in Figure 1. We found that unlike cancer genes (discussed above), disease-related genes are more or less evenly distributed across all categories with different baseline tolerance. As a result, the original scores provided by the tools for SNVs within these genes are transformed in either direction, with no clear resulting trend. (In the case of cancer genes, the scores of their SNVs generally tend to become amplified upon transformation, because they usually appear in lowly tolerant classes.) Nevertheless, we also observed that groups with low tolerance tend to be enriched in dominant disease genes, while the opposite occurs with recessive disease genes. Therefore, we hypothesize that, upon transformation, the FISs of nsSNVs in dominant disease genes increase, but those in recessive disease genes decrease, making them similar to neutral variants.

### Implementation of the method

The approach we have described to transform well-established FIS calculations to take into account the differences in baseline tolerance to nsSNVs between protein families can be easily implemented. It is important to highlight that although we have used SIFT, PPH2, MA and CHASM to present and test our approach, in principle this transformation can be applied to any other FIS.

The best overall performance in the classification of the nine proxy datasets was achieved by the transformed FIS based on the GOMF. Therefore, we decided to follow that classification system for implementation of our transFIC, as well as for the web server.

Note that the inferior limit of 20 SNVs to compute the baseline tolerance (described in the 'Detecting differences in baseline tolerance across genes' section) applies not to a single gene but rather to the SNVs pooled from genes within the same functional group(s). With this limit, we were able to successfully transform the FISs of nsSNVs in 15,651 genes using the GOBP classification scheme, 17,229 genes using GOMF, 11,642 using Doms and 6,830 using CPs. For nsSNVs in the remaining genes - which are either not classified within a given system, or do not belong to groups that account for at least 20 SNVs - we compute a transFIC using the mean and standard deviation of all the SNVs in the 1000 Genomes Project.

### Interpretation of transFIC scores

To facilitate the interpretation of transFIC SIFT, PPH2 and MA results, we have devised three categories (low, medium and high impact) into which somatic mutations can be classified based on their transformed FIS. For each transFIC, complementary cumulative distributions of non-recurrent, recurrent and highly recurrent COSMIC mutations were taken into account in defining the categories, an idea that we adapted from the MA tool [3].

**Figure 4:**
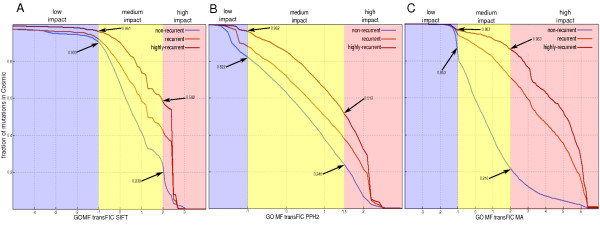
**Complementary cumulative distribution of the three transFIC of subsets of nonsynonymous single nucleotide variants from COSMIC**. **(a-c) **Complementary cumulative distribution of transFIC SIFT (a), transFIC PPH2 (b) and transFIC MA (c) of nonrecurrent (blue), recurrent (orange) and highly recurrent (red) COSMIC mutations.

The boundaries of these categories were defined as follows: low impact upper boundary (SIFT -1, PPH2 -1, MA -1), drawn at the transFIC score above which lays approximately 95% of the distribution of highly recurrent COSMIC mutations (in other words, this category contains at most approximately 5% of highly recurrent COSMIC mutations); high impact lower boundary (SIFT 2, PPH2 1.5, MA 2), a transFIC cutoff establishing a category with at most approximately 25% of the distribution of nonrecurrent COSMIC mutations; and medium impact, the remaining mutations with transFIC scores between these two limits. The concept of this categorization, as well as the categories themselves for the three transFIC presented here, are illustrated in Figure 4a-c. The specificity and sensitivity attained by the transFIC of the three tools at separating highly recurrent from non-recurrent COSMIC mutations and recurrent from non-recurrent COSMIC mutations at each of these cutoffs are presented in Additional file 9.

The results shown in Figure 4 (as well as those in Figure 3) reveal that the MA transFIC exhibits the best performance amongst the three transformed scores in the transFIC website to distinguish between highly recurrent and non-recurrent COSMIC mutations. Nevertheless, we believe that it is important for the researcher to appraise the three transFIC scores of their mutations to make an informed decision regarding the likely functional impact of their somatic mutations. Also, it is important to bear in mind that the researcher may replicate the approach described in this paper to transform any other score of functional impact of SNVs to produce their own transFIC.

## Conclusions

We observed large differences in the FIS distribution of nsSNVs from different protein groups, which indicates that genes with distinct functions possess a different baseline tolerance to deleterious mutations. We exploited these differences of baseline tolerance to transform the FISs of cancer somatic mutations provided by three well-known bioinformatics tools. The transformed FIS systematically outperforms the original FIS on nine proxy validation sets, each composed of a positive set of mutations enriched in driver nsSNVs and a negative set of mutations enriched in passenger nsSNVs (or polymorphisms).

Therefore, we recommend the use of a transformed FIS to assess the functional impact of cancer mutations. We have implemented the method to compute the transformed FIS of these three tools, which we call transFIC (transformed Functional Impact Scores in Cancer). We distribute it as a PERL script that users can download and use locally. We have also set up a web server that can be queried to obtain the transFIC of somatic cancer nsSNVs.

## Abbreviations

ACC: accuracy; CGC: Cancer Gene Census; COSMIC: Catalog of Somatic Mutations in Cancer; CP: MSigDB canonical pathway; Dom: Pfam Domain; FIS: functional impact score; GOBP: Gene Ontology Biological Process; GOMF: Gene Ontology Molecular Function; ICGC: International Cancer Genome Consortium; MA: MutationAssessor; MCC: Matthew's correlation coefficient; nsSNV: nonsynonymous single nucleotide variant; PPH2: PolyPhen2; SNV: single nucleotide variant; WG: whole genome.

## Competing interests

The authors declare that they have no competing interests.

## Authors' contributions

AGP carried out the study and contributed to drafting and editing the manuscript. NLB supervised the study and contributed to drafting and editing the manuscript. JDP configured and set up the transFIC webserver. All authors read and approved the final manuscript.

## Supplementary Material

Additional file 1 **A graph depicting the distribution of FIS of nsSNVS in groups of genes that belong to different canonical pathways**. The graph is analogous to Figure 1. Click here for file

Additional file 2 **A graph depicting the distribution of FISs of nsSNVs in groups of genes that belong to different GOBPs**. The graph is analogous to Figure 1. Click here for file

Additional file 3 **A graph depicting the distribution of FISs of nsSNVs in groups of genes with different Pfam domains**. The graph is analogous to Figure 1. Click here for file

Additional file 4 **Comparison of the mean MA score assigned to each gene with the approach explained in the main paper using the GOBP and the GOMF classifications**. Click here for file

Additional file 5 **A table with the results of comparing the prevalence of cancer genes versus non-cancer genes (and tumor suppresor genes versus oncogenes) amongst those that belong to the 100 with lowest baseline tolerance and the 100 with the highest baseline tolerance in the GOMF category**. Click here for file

Additional file 6 **Tables and figures showing the Matthew's correlation coefficients and overall accuracy of transformed FISs on the nine proxy datasets**. This is the same data presented in Figure 3. It also contains discussion on observations comparing SIFT, PPH2 and MA improvements with transFIC in different datasets. Click here for file

Additional file 7 **A table and figure showing the performance of the four methods used (SIFT, PPH2, MA and CHASM) and its transFIC scores (GOMF) in the classification of two proxy datasets and a modified version of them excluding mutations used to train CHASM**. Click here for file

Additional file 8 **A table showing the Matthew's correlation coefficients and overall accuracy of transformed FISs on a dataset of disease-related nsSNVs and polymorphisms**. Click here for file

Additional file 9 **Two tables showing the values of sensitivity and specificity attained by the transFIC of the three methods when separating highly recurrent from non-recurrent COSMIC mutations and recurrent from non-recurrent COSMIC mutations**. Click here for file
