# Supplementary material for: Improving the prediction of the functional impact of cancer mutations by baseline tolerance transformation
Source: Genome Med. 2012 Nov 26;4(11):89. doi: 10.1186/gm390 (PMC4064314; doi:10.1186/gm390)
Supplement: Additional file 1 — A graph depicting the distribution of FIS of nsSNVS in groups of genes that belong to different canonical pathways. The graph is analogous to Figure 1. [file gm390-S1.PDF]

Additional File 1

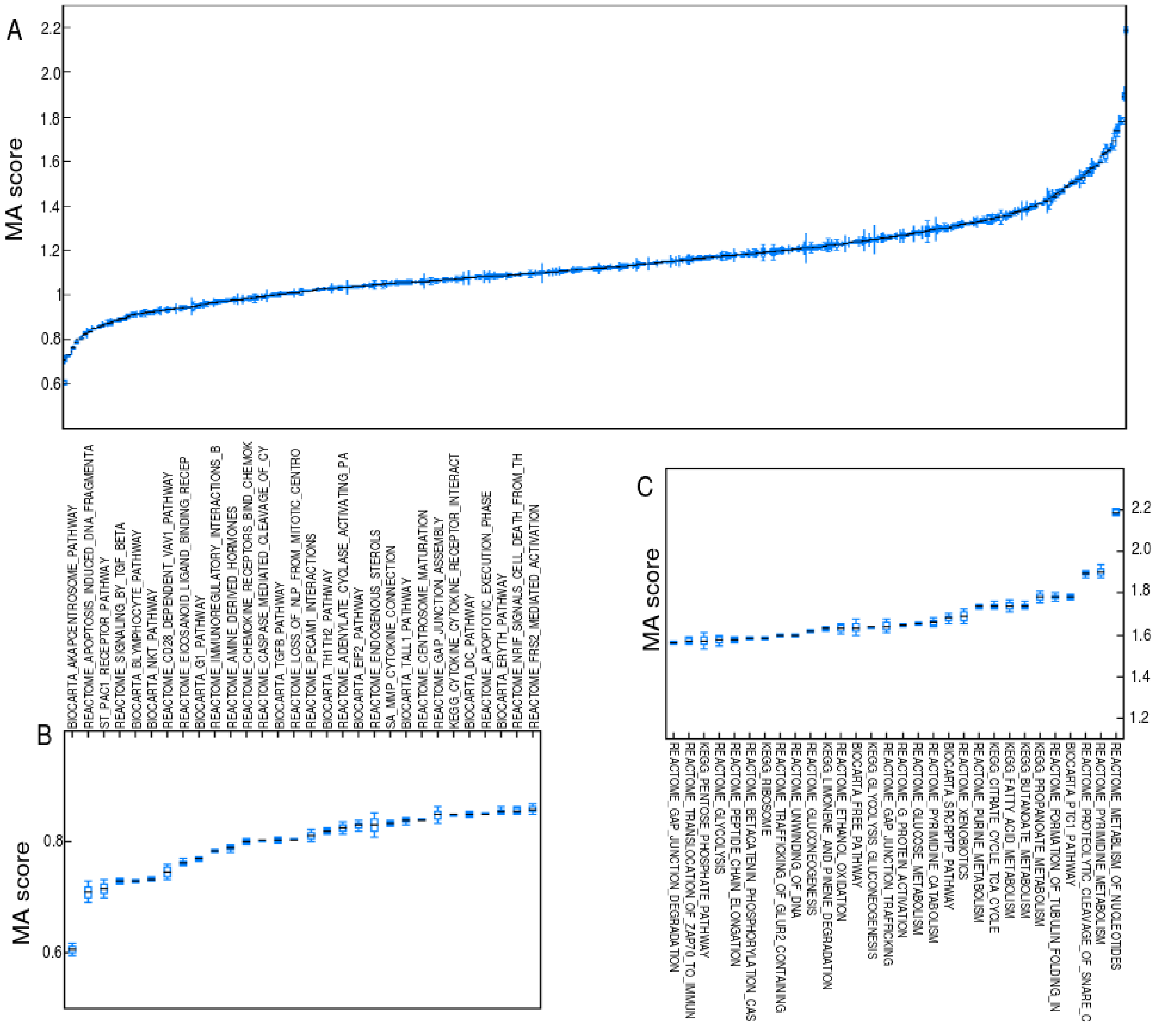

The MutationAssessor (MA) scores of germline SNVs are differentially distributed across canonical pathways (CP). A, B and C. Candlesticks representation of the distribution of MA scores of SNVs in all CP, ordered by their MA score means (A), the thirty CP at the lower end (B) and the thirty CP at the upper end (C) of the MA score spectrum. The line at the center of the candlesticks correspond to the mean of the distribution; the box around it are delimited by the value of the mean minus/plus one standard error of the mean, and the whiskers extend out to the mean minus/plus two standard errors of the mean. (Pathways' names are truncated.)
