# Supplementary material for: Improving the prediction of the functional impact of cancer mutations by baseline tolerance transformation
Source: Genome Med. 2012 Nov 26;4(11):89. doi: 10.1186/gm390 (PMC4064314; doi:10.1186/gm390)
Supplement: Additional file 2 — A graph depicting the distribution of FISs of nsSNVs in groups of genes that belong to different GOBPs. The graph is analogous to Figure 1. [file gm390-S2.PDF]

Additional File 2

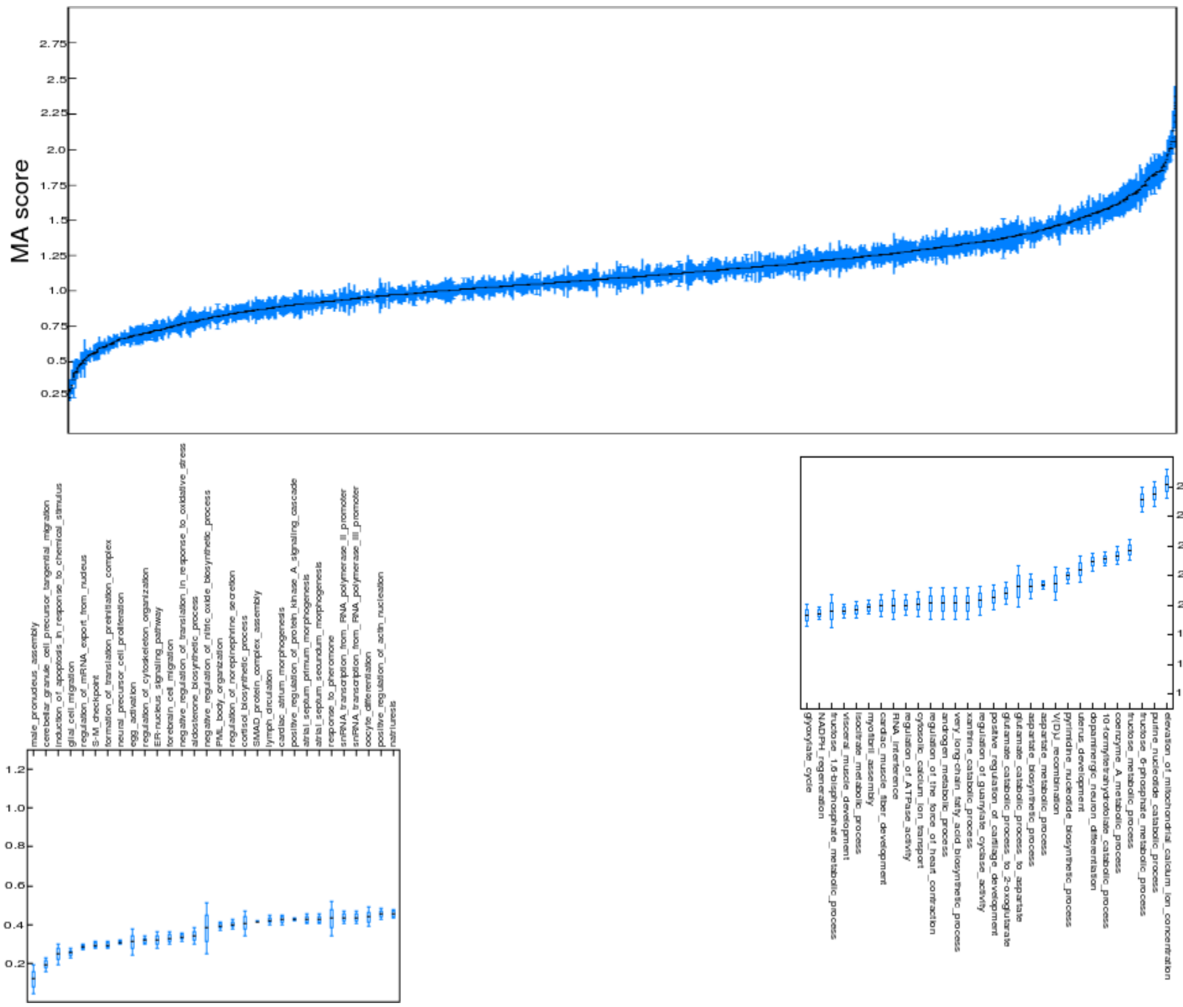

The MutationAssessor (MA) scores of germline SNVs are differentially distributed across Gene Ontologies Biological Processes (GOBP). A, B and C. Candlesticks representation of the distribution of MA scores of SNVs in all GOBP (A), the thirty GOBP at the lower end (B) and the thirty GOBP at the upper end (C) of the MA score spectrum. (GOBP names are truncated.)
