# Supplementary material for: Improving the prediction of the functional impact of cancer mutations by baseline tolerance transformation
Source: Genome Med. 2012 Nov 26;4(11):89. doi: 10.1186/gm390 (PMC4064314; doi:10.1186/gm390)
Supplement: Additional file 3 — A graph depicting the distribution of FISs of nsSNVs in groups of genes with different Pfam domains. The graph is analogous to Figure 1. [file gm390-S3.PDF]

Additional File 3

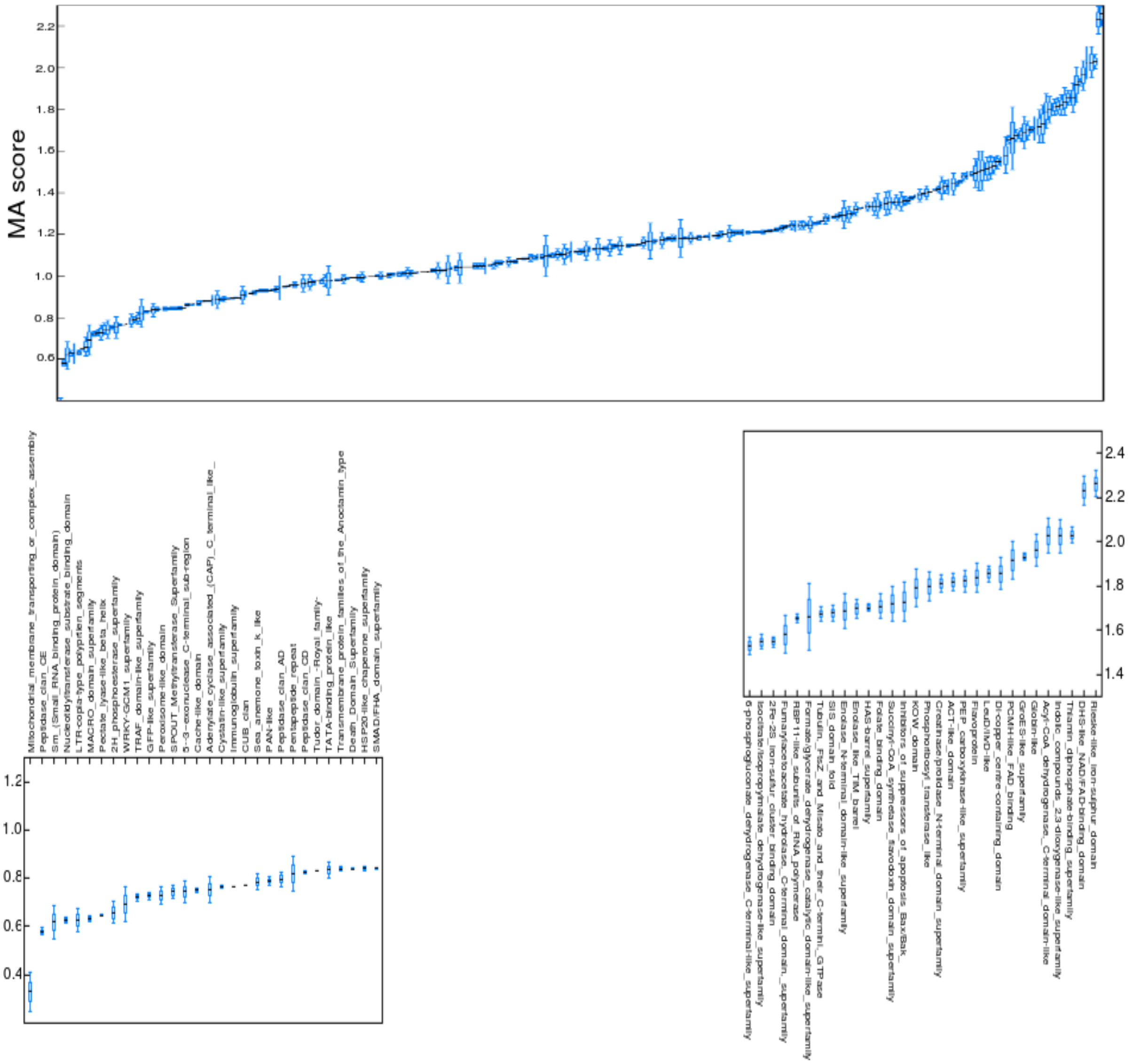

The MutationAssessor (MA) scores of germline SNVs are differentially distributed across Pfam domains (Doms). A, B and C. Candlesticks representation of the distribution of MA scores of SNVs in all Doms (A), the thirty Doms at the lower end (B) and the thirty Doms at the upper end (C) of the MA score spectrum. (Domain names are truncated.)
