# Supplementary material for: Improving the prediction of the functional impact of cancer mutations by baseline tolerance transformation
Source: Genome Med. 2012 Nov 26;4(11):89. doi: 10.1186/gm390 (PMC4064314; doi:10.1186/gm390)

Additional File 4

Comparison of the mean MA score assigned to each gene with the approach explained in the main paper using the GOBP and the GOMF classifications.

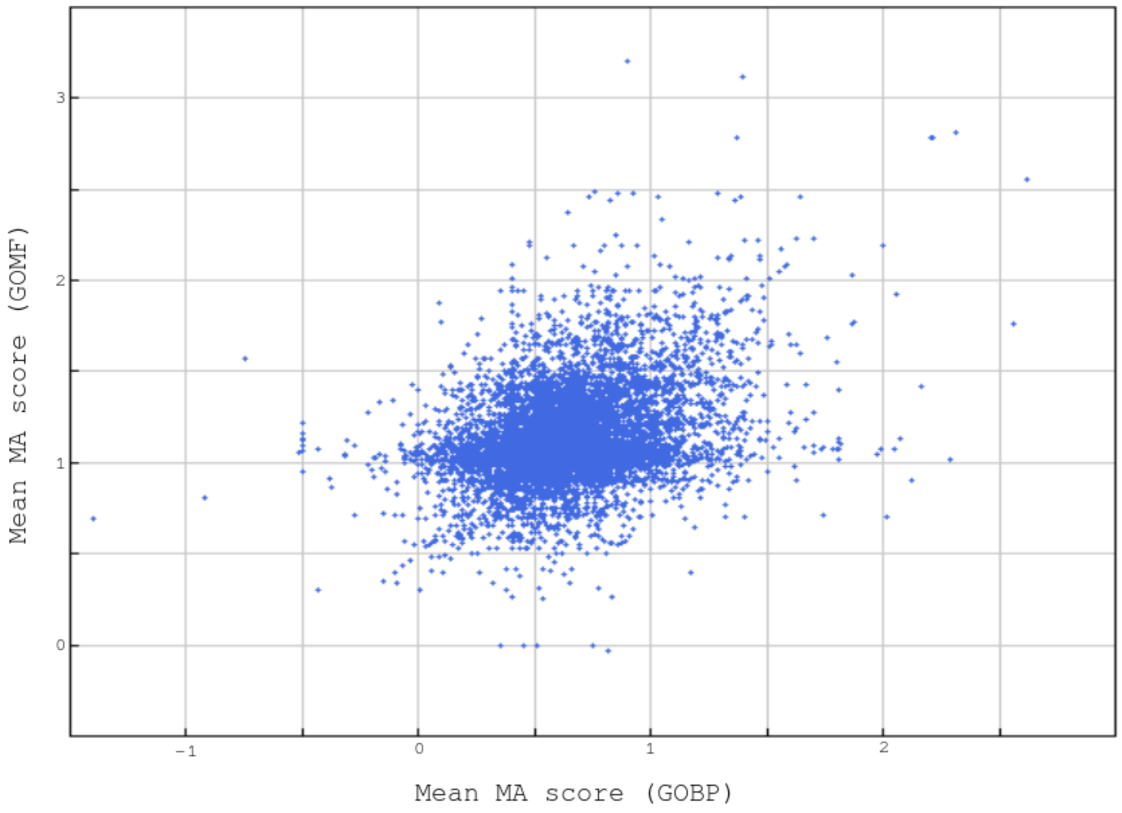

Supplement: Additional file 4 — Comparison of the mean MA score assigned to each gene with the approach explained in the main paper using the GOBP and the GOMF classifications. [file gm390-S4.PDF]
