# Supplementary material for: Improving the prediction of the functional impact of cancer mutations by baseline tolerance transformation
Source: Genome Med. 2012 Nov 26;4(11):89. doi: 10.1186/gm390 (PMC4064314; doi:10.1186/gm390)
Supplement: Additional file 5 — A table with the results of comparing the prevalence of cancer genes versus non-cancer genes (and tumor suppresor genes versus oncogenes) amongst those that belong to the 100 with lowest baseline tolerance and the 100 with the highest baseline tolerance in the GOMF category. [file gm390-S5.PDF]

Additional File 5

Comparison of the prevalence of cancer genes/non-cancer genes (and tumor suppresor genes/oncogenes) amongst those that belong to the 100 GOs (molecular function) with lowest basal tolerance and the 100 GOs with highest baseline tolerance. Cancer genes were extracted from the Cancer Gene Census.

	Lowest tolerance 100 GOMF groups	Highest tolerance 100 GOMF groups	P-value (Fisher's test)
Cancer genes	35	15	0.0012
Non-cancer genes	397	461	
Tumor suppressor genes	14	6	non significant
Oncogenes	21	10	
