# Supplementary material for: Improving the prediction of the functional impact of cancer mutations by baseline tolerance transformation
Source: Genome Med. 2012 Nov 26;4(11):89. doi: 10.1186/gm390 (PMC4064314; doi:10.1186/gm390)
Supplement: Additional file 6 — Tables and figures showing the Matthew's correlation coefficients and overall accuracy of transformed FISs on the nine proxy datasets. This is the same data presented in Figure 3. It also contains discussion on observations comparing SIFT, PPH2 and MA improvements with transFIC in different datasets. [file gm390-S6.PDF]

Additional File 6

A) Performance of transformed SIFT score (transFIC SIFT) compared to the original SIFT at classifying the proxy datasets

	SIFT													
	GOBP			GOMF			Doms			CP			Original	
	MCC	ACC	Cutoff	MCC	ACC	Cutoff	MCC	ACC	Cutoff	MCC	ACC	Cutoff	MCC	ACC
Cosmic5+/1	0.16	0.74	2.3	0.16	0.77	2.2	0.19	0.79	1.9	0.16	0.73	2.4	0.12	0.49
Cosmic2+/1	0.14	0.72	2.3	0.14	0.74	2.2	0.16	0.77	1.9	0.14	0.71	3.8	0.1	0.49
Cosmic5+/Pol	0.78	0.97	1.8	0.73	0.96	1.8	0.75	0.96	1.8	0.43	0.87	1.3	0.32	0.71
Cosmic2+/Pol	0.66	0.9	1.7	0.61	0.89	1.7	0.61	0.89	1.8	0.41	0.82	1.3	0.32	0.7
CosmicD/O	0.13	0.73	2.2	0.11	0.68	2.2	0.1	0.66	1.8	0.12	0.72	2	0.09	0.48
CosmicD/Pol	0.72	0.95	1.7	0.64	0.94	1.7	0.55	0.93	1.8	0.38	0.86	1.3	0.29	0.7
CosmicCGC/nonCGC	0.24	0.66	2.3	0.22	0.66	2.2	0.17	0.62	1.9	0.17	0.62	2	0.08	0.44
WG2+/WG1	0.05	0.82	2.3	0.1	0.92	2.1	0.04	0.76	1.9	0.04	0.78	2.1	0.02	0.84
WGCGC/nonCGC	0.19	0.79	2.1	0.22	0.83	2.1	0.16	0.71	1.9	0.17	0.75	2.1	0.11	0.42

GOBP: transFIC obtained using the distribution of SIFT scores of germline SNVs in genes with different GO biological processes
GOMF: transFIC obtained using the distribution of SIFT scores of germline SNVs in genes with different GO molecular function
Doms: transFIC obtained using the distribution of SIFT scores of germline SNVs in genes with different Pfam domains
CP: transFIC obtained using the distribution of SIFT scores of germline SNVs in genes with different canonical pathways

MCC: Matthew's correlation coefficient
ACC: Overall accuracy
Cutoff: TransFIC value that maximizes the MCC

B) Performance of transformed PPH2 score (transFIC PPH2) compared to the original PPH2 at classifying the proxy datasets

	PPH2													
	GOBP			GOMF			Doms			CP			Original	
	MCC	ACC	Cutoff	MCC	ACC	Cutoff	MCC	ACC	Cutoff	MCC	ACC	Cutoff	MCC	ACC
Cosmic5+/1	0.23	0.94	2.7	0.22	0.93	2.1	0.13	0.82	1.9	0.26	0.94	2.2	0.09	0.6
Cosmic2+/1	0.2	0.9	2.7	0.19	0.89	2.1	0.11	0.78	1.9	0.24	0.9	2.4	0.06	0.59
Cosmic5+/Pol	0.44	0.9	1.7	0.44	0.9	1.6	0.45	0.91	1.3	0.5	0.92	1.4	0.41	0.86
Cosmic2+/Pol	0.39	0.81	1.6	0.4	0.78	0.3	0.39	0.81	1.1	0.44	0.84	1.4	0.39	0.79
CosmicD/O	0.18	0.93	2.2	0.19	0.91	2	0.13	0.81	1.8	0.24	0.93	2	0.1	0.61
CosmicD/Pol	0.46	0.89	1.4	0.47	0.89	1.6	0.45	0.9	1.1	0.52	0.9	1.5	0.42	0.85
CosmicCGC/nonCGC	0.24	0.66	2.2	0.22	0.66	2	0.15	0.64	1.9	0.24	0.66	1.9	0.07	0.56
WG2+/WG1	0.07	0.94	2.5	0.08	0.94	2.1	0.08	0.95	1.9	0.12	0.96	2.2	0.01	0.71
WGCGC/nonCGC	0.29	0.92	2.3	0.24	0.91	2.1	0.22	0.91	1.9	0.31	0.92	2.2	0.11	0.56

GOBP: transFIC obtained using the distribution of PPH2 scores of germline SNVs in genes with different GO biological processes
GOMF: transFIC obtained using the distribution of PPH2 scores of germline SNVs in genes with different GO molecular function
Doms: transFIC obtained using the distribution of PPH2 scores of germline SNVs in genes with different Pfam domains
CP: transFIC obtained using the distribution of PPH2 scores of germline SNVs in genes with different canonical pathways

MCC: Matthew's correlation coefficient
ACC: Overall accuracy
Cutoff: TransFIC value that maximizes the MCC

C) Performance of transformed MA score (transFIC MA) compared to the original MA at classifying the proxy datasets

	GOBP			GOMF			Doms			CP			Original	
	MCC	ACC	Cutoff	MCC	ACC	Cutoff	MCC	ACC	Cutoff	MCC	ACC	Cutoff	MCC	ACC
Cosmic5+/1	0.33	0.91	2	0.57	0.97	4.6	0.3	0.92	2.2	0.43	0.95	3.1	0.32	0.9
Cosmic2+/1	0.3	0.87	1.9	0.5	0.93	3.8	0.28	0.78	2.1	0.37	0.91	2.2	0.3	0.8
Cosmic5+/Pol	0.78	0.97	1.1	0.86	0.98	2.2	0.74	0.95	1	0.79	0.97	2.2	0.8	0.96
Cosmic2+/Pol	0.7	0.9	1	0.76	0.93	1,6	0.68	0.9	1	0.69	0.91	1.3	0.71	0.91
CosmicD/O	0.19	0.88	2.3	0.25	0.88	2.3	0.17	0.67	1	0.19	0.92	3.2	0.18	0.78
CosmicD/Pol	0.63	0.93	1.1	0.69	0.94	1.1	0.59	0.91	1	0.58	0.92	1.4	0.64	0.92
CosmicCGC/nonCGC	0.26	0.68	1.1	0.5	0.85	3.4	0.27	0.69	1	0.3	0.8	2.5	0.16	0.78
WG2+/WG1	0.08	0.93	2	0.23	0.96	4.6	0.07	0.91	2.2	0.12	0.95	2.7	0.1	0.89
WGCGC/nonCGC	0.32	0.86	2	0.52	0.94	4.4	0.36	0.9	2.2	0.38	0.91	2.7	0.34	0.9

GOBP: transFIC obtained using the distribution of MA scores of germline SNVs in genes with different GO biological processes
GOMF: transFIC obtained using the distribution of MA scores of germline SNVs in genes with different GO molecular function
Doms: transFIC obtained using the distribution of MA scores of germline SNVs in genes with different Pfam domains
CP: transFIC obtained using the distribution of MA scores of germline SNVs in genes with different canonical pathways

MCC: Matthew's correlation coefficient
ACC: Overall accuracy
Cutoff: TransFIC value that maximizes the MCC

D) Performance of GOBP transFIC compared to original FIS at classifying the eight proxy datasets.

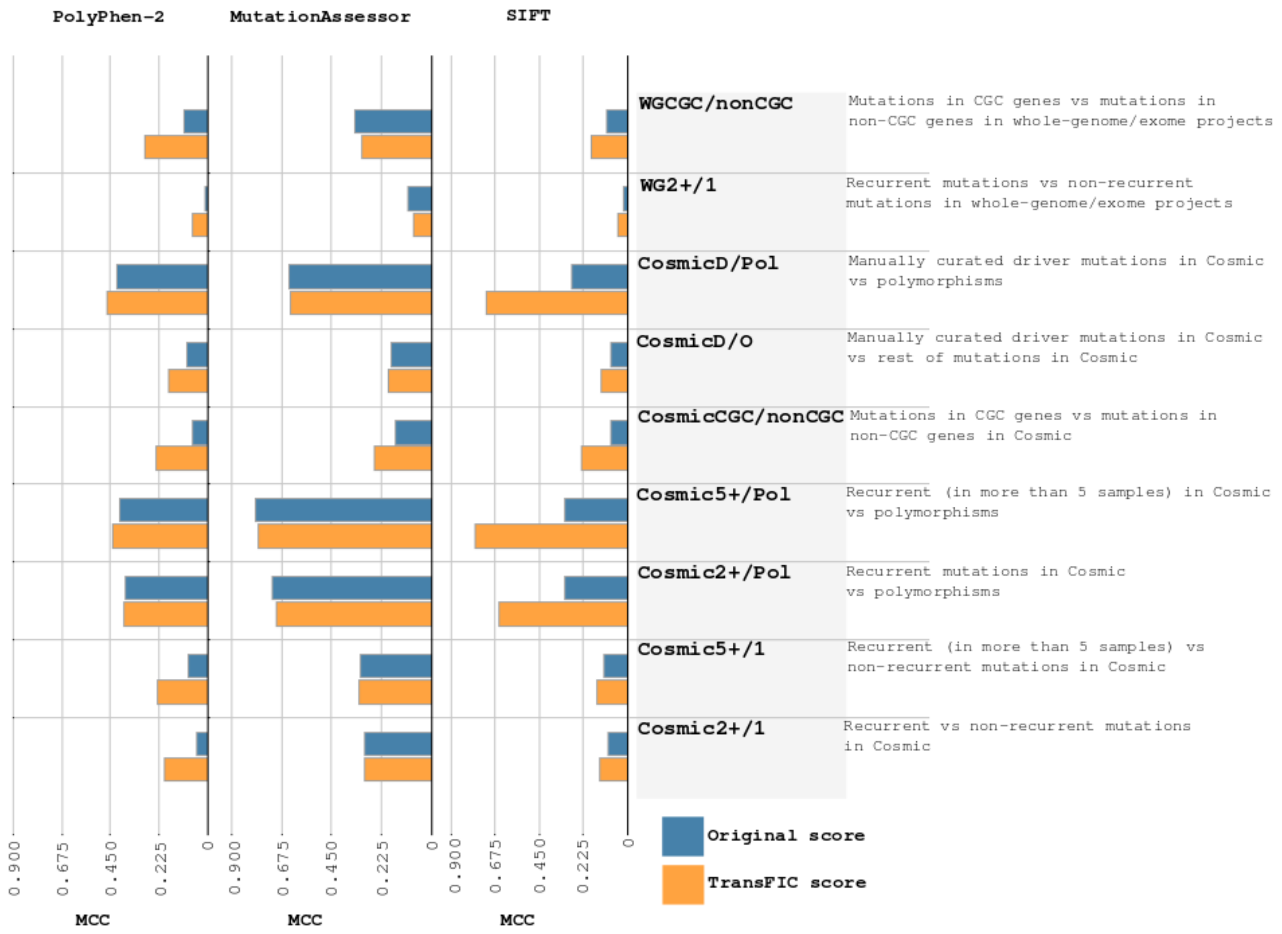

E) Performance of CP transFIC compared to original FIS at classifying the eight proxy datasets.

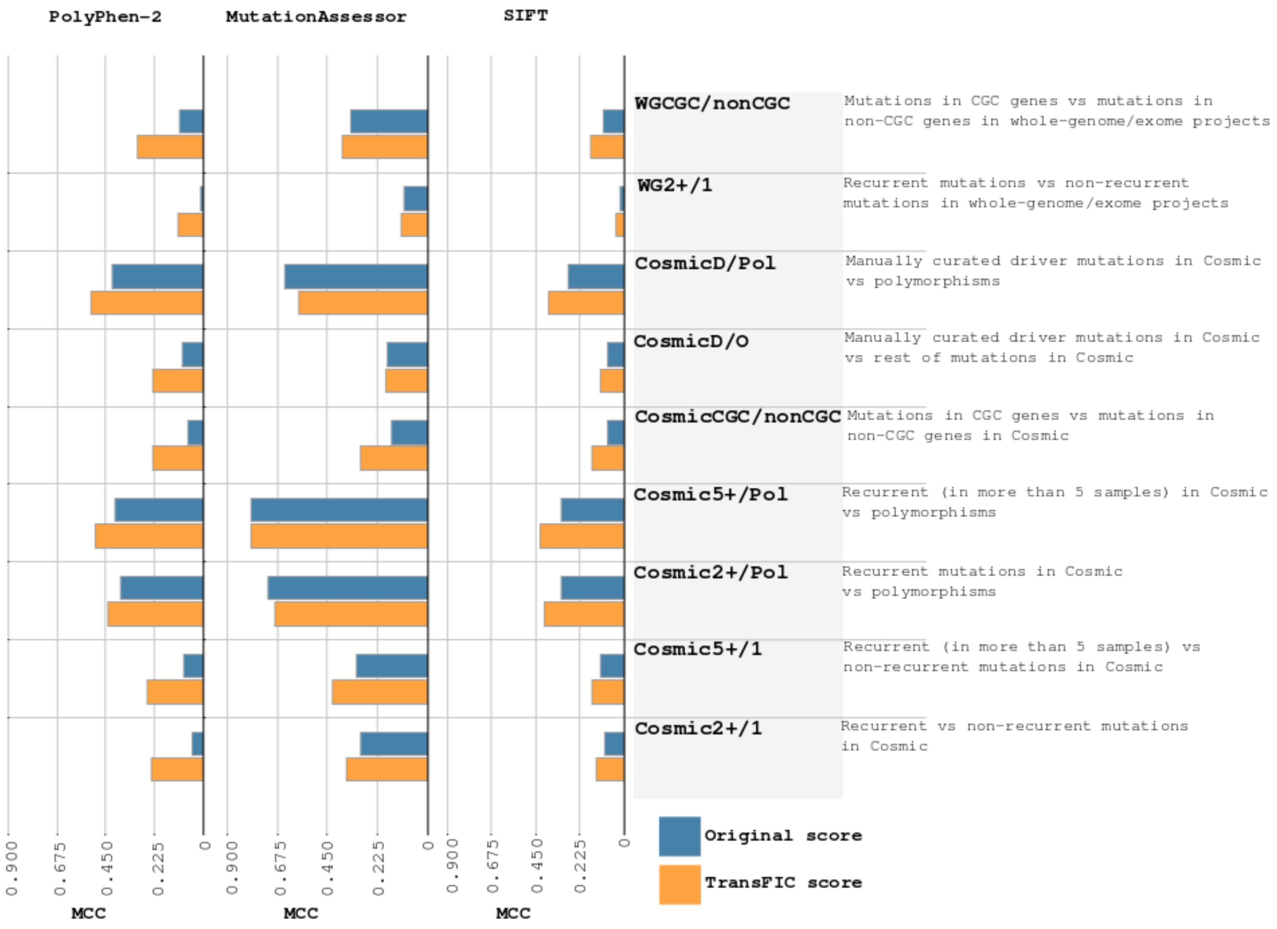

F) Performance of Doms transFIC compared to original FIS at classifying the eight proxy datasets.

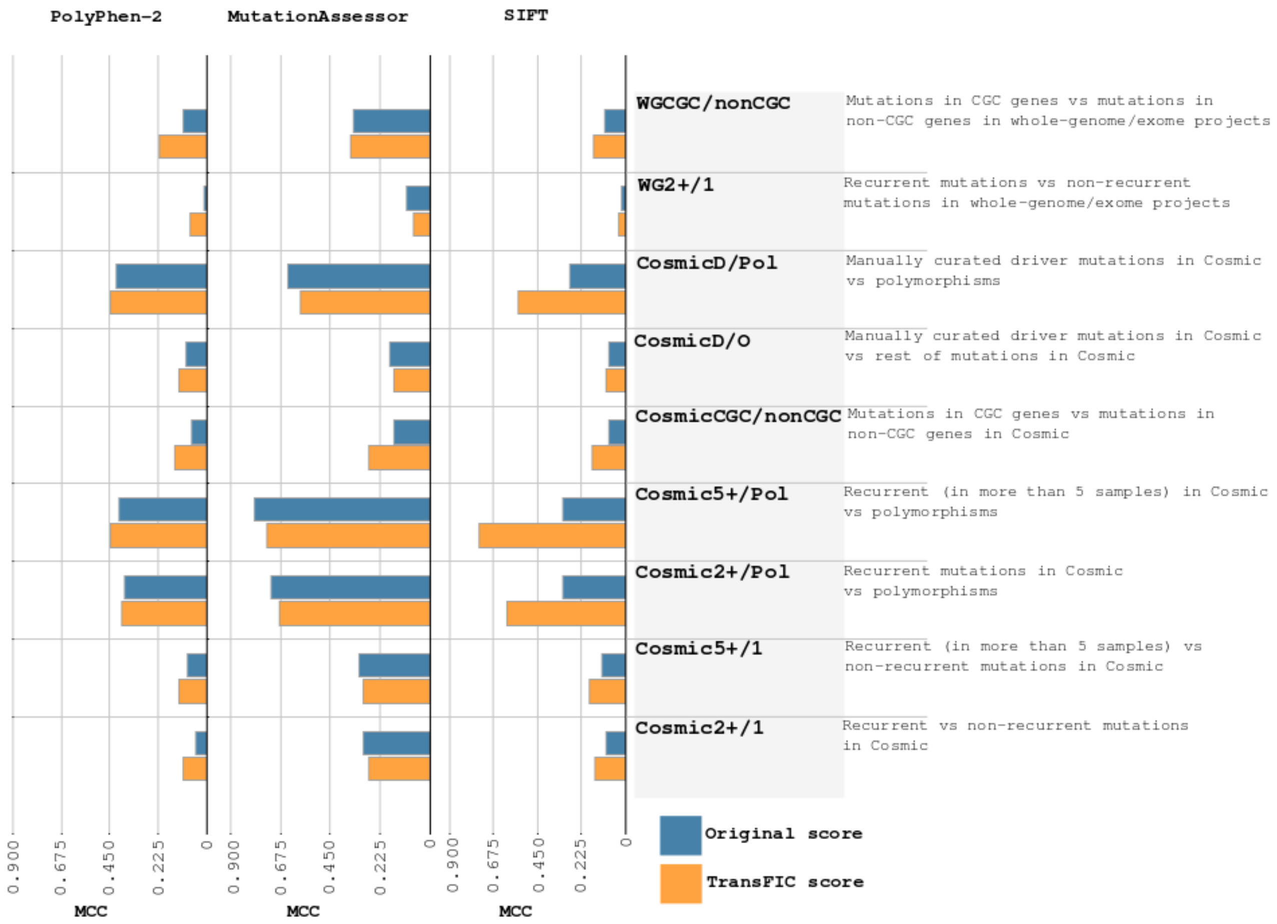

G) Observations comparing SIFT, PPH2 and MA improvements with transFIC in different datasets

One observation that arises from Figure 3 is that the transFIC of SIFT attains the most significant improvement in datasets whose negatives subset is composed of polymorphisms, while those of PPH2 and MA show a greater gain where the negatives subset contains non-recurrent mutations. (Compare, for instance the results with Cosmic5+/Pol and with Cosmic5+/1.) We think it's easier to understand the reason for this if the accuracies attained by the transFIC and the original scores for these datasets are observed in addition to the MCCs (additional file 6). For example, the MCC gain for the transFIC of SIFT is 0.407 (0.320-0.727) in the Cosmic5+/Pol dataset, and 0.04 (0.122-0.162) in Cosmic5+/1. However, the corresponding accuracy gains are very similar: 0.249 and 0.278, respectively. The same thing, in the opposite direction occurs with the transFIC of MA. While the MCC gains in the datasets are 0.062 and 0.243, the accuracy gains are much more similar: 0.013 and 0.067. Therefore, in these two cases the differences in MCC gains probably occur due to different proportions of cases being misclassified, but which altogether produce comparable global accuracy gains. It is important to bear in mind that the MCC is much more sensitive to the amount of false positive and false negative instances than the accuracy.

In the case of PPH2, bot the MCC and the accuracy experience very small gains in the Cosmic5+/Pol dataset (0.036 and 0.039, respectively), but substantially larger gains in the Cosmic5+/1 datasets (0.127 and 0.322). Probably, the performance of PPH2 cannot be significantly improved by the transFIC in datasets whose negatives subset are composed of polymorphisms which have been extracted from the HumVar dataset that was used to test PPH2 in the first place. To test this last point, we replaced the polymorphisms in the CosmicD/Pol, Cosmic5+/Pol, and Cosmic2+/Pol by other neutral SNVs, obtained from dbSNP and curated by Thusberg *et al.*, 2011 (ref. 6 in the main paper). (We had previously removed from this dataset the SNVs present in HumVar, after which the dataset contained 6265 SNVs scored by the three methods.) We then re-computed the MCC and accuracy attained by

PPH2 and the transFIC of PPH2 in these three modified proxy datasets. The results are presented in table H below, and show that while transFIC PPH2 exhibit comparable performance (MCC and accuracy) in the original and modified proxy datasets, the performance of the original PPH2 score is definitely smaller in the modified proxy datasets. This different behavior accounts for a gain of 0.146 in MCC in the Cosmic5+/Pol modified proxy dataset, compared to 0.034 in the homologous original dataset.

H) Performance of the transFIC PPH2 score compared to the original PPH2 at classifying the three modified proxy datasets (see above). (Its performance in the original proxy datasets is also shown for comparative purposes.)

	GOBP		GOMF		Doms		CP		Original	
	MCC	ACC	MCC	ACC	MCC	ACC	MCC	ACC	MCC	ACC
Cosmic5+/Polymorphisms (original)	0.44	0.903	0.442	0.899	0.448	0.913	0.5	0.915	0.406	0.86
Cosmic5+/Polymorphisms (modif.)	0.438	0.893	0.458	0.908	0.447	0.93	0.492	0.934	0.292	0.706
Cosmic2+/Polymorphisms (original)	0.391	0.805	0.402	0.78	0.394	0.813	0.443	0.839	0.385	0.787
Cosmic2+/Polymorphisms (modif.)	0.385	0.797	0.393	0.842	0.362	0.831	0.416	0.848	0.277	0.729
Chasm_drivers/Polymorphisms (original)	0.463	0.89	0.466	0.887	0.448	0.895	0.516	0.902	0.424	0.852
Chasm_drivers/Polymorphisms (modif)	0.443	0.899	0.457	0.901	0.411	0.894	0.481	0.911	0.319	0.772
