# Supplementary material for: Improving the prediction of the functional impact of cancer mutations by baseline tolerance transformation
Source: Genome Med. 2012 Nov 26;4(11):89. doi: 10.1186/gm390 (PMC4064314; doi:10.1186/gm390)
Supplement: Additional file 7 — A table and figure showing the performance of the four methods used (SIFT, PPH2, MA and CHASM) and its transFIC scores (GOMF) in the classification of two proxy datasets and a modified version of them excluding mutations used to train CHASM. [file gm390-S7.PDF]

Additional File 7

As explained in the main paper, we computed the transFIC (GOMF) of CHASM and we evaluated the performance of the original and transFIC scores using two datasets, namely WG2+/1 and the WGCGC/nonCGC. Since CHASM was trained with manually curated driver mutations from COSMIC we could not assess the performance of CHASM in those datasets. Also the evaluation was performed on a modified version of the two aforementioned proxy datasets, lacking the mutations that appear within the training set of CHASM (see Methods section in the main paper). To be able to compare the performance of different methods we computed Matthews Correlation Coefficient (MCC) and Accuracy (ACC) for all the methods (SIFT, PPH2, MA and CHASM) with the original (WG2+/1 and WGCGC/nonCGC) and modified datasets (WG2+/1* and WGCGC/nonCGC*).

The results of this assessment are illustrated in Figure 1 and the Table 1 below. It is important to observe that the classification of the datasets by CHASM is based on the qvalues provided by the CHASM tools rather than on the actual CHASM score. We represent in Figure 1 the qvalue cutoff that maximizes MCC (qvalue<0.05).

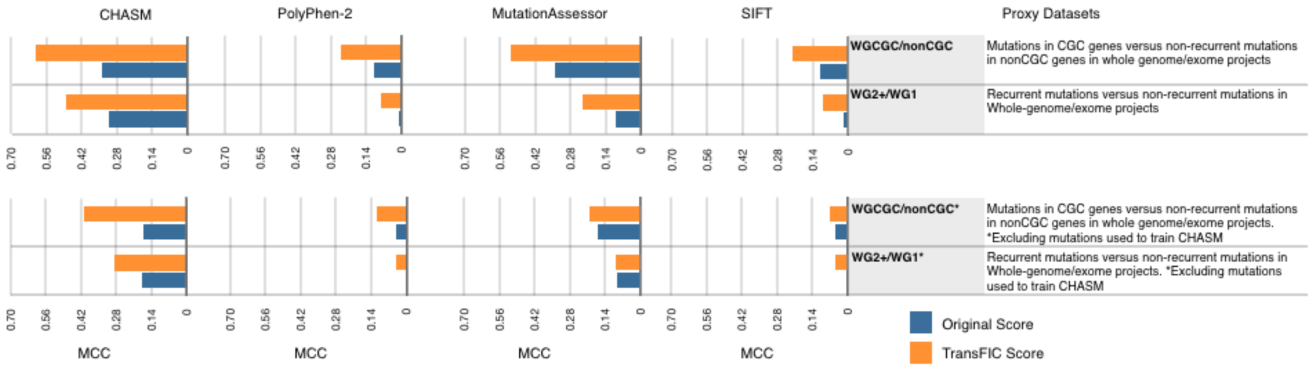

Figure 1. Transformed Functional Impact for Cancer (TransFIC) systematically outperforms original scores in the task of differentiating cancer driver mutations from neutral variants. Performance of GOMF transFIC is compared to the four original functional impact scores (FIS) classifying the four proxy datasets, using as cutoff the value of FIS (or transFIC) that maximizes the Mathews Correlation Coefficient (MCC) in each case, with the exception of CHASM in which the qvalue cutoff <0.05 is used for classification (qvalue cutoff the maximizes MCC).

Table 1
Performance of GOMF transFIC the four original functional impact scores (FIS) classifying the four proxy datasets.

	SIFT							
	transFIC				original			
	MCC	ACC	SENS	SPEC	MCC	ACC	SENS	SPEC
WG2+/WG1	0.1	0.92	0.41	0.94	0.02	0.84	0.68	0.84
WG2+/WG1*	0.05	0.71	0.32	0.75	-0.02	0.4	0.59	0.38
WGCGC/WGnonCGC	0.22	0.83	0.55	0.84	0.11	0.42	0.7	0.41
WGCGC/WGnonCGC*	0.07	0.58	0.58	0.58	0.05	0.41	0.72	0.39
	PPH2							
	transFIC				original			
	MCC	ACC	SENS	SPEC	MCC	ACC	SENS	SPEC
WG2+/WG1	0.23	0.96	0.11	1	0.1	0.89	0.51	0.89
WG2+/WG1*	0.04	0.89	0.06	0.97	-0.02	0.53	0.41	0.55
WGCGC/WGnonCGC	0.24	0.91	0.18	0.94	0.11	0.56	0.24	0.58
WGCGC/WGnonCGC*	0.12	0.88	0.12	0.97	0.04	0.6	0.48	0.6
	MA							
	transFIC				original			
	MCC	ACC	SENS	SPEC	MCC	ACC	SENS	SPEC
WG2+/WG1	0.23	0.96	0.36	0.99	0.1	0.89	0.28	0.91
WG2+/WG1*	0.1	0.84	0.2	0.91	0.09	0.87	0.13	0.95
WGCGC/WGnonCGC	0.52	0.94	0.52	0.95	0.34	0.9	0.41	0.92
WGCGC/WGnonCGC*	0.2	0.88	0.35	0.92	0.17	0.87	0.26	0.93
	CHASM							
	transFIC				Original (0.005)			
	MCC	ACC	SENS	SPEC	MCC	ACC	SENS	SPEC
WG2+/WG1	0.48	0.92	0.31	0.94	0.04	0.85	0.09	0.94
WG2+/WG1*	0.29	0.93	0.12	1	0.06	0.87	0.11	0.94
WGCGC/WGnonCGC	0.6	0.94	0.43	0.96	-0.04	0.86	0.03	0.94
WGCGC/WGnonCGC*	0.41	0.95	0.24	1	-0.02	0.89	0.04	0.94
	CHASM							
	Original (0.01)				Original (0.05)			
	MCC	ACC	SENS	SPEC	MCC	ACC	SENS	SPEC
WG2+/WG1	0.31	0.87	0.34	0.88	0.31	0.87	0.35	0.88
WG2+/WG1*	0.17	0.88	0.22	0.94	0.18	0.88	0.22	0.94
WGCGC/WGnonCGC	0.34	0.89	0.4	0.89	0.34	0.89	0.4	0.87
WGCGC/WGnonCGC*	0.16	0.9	0.23	0.94	0.17	0.9	0.23	0.94

MCC: Matthews Correlation Coefficient
ACC: Accuracy
SENS: Sensitivity
SPEC: Specificity
CHASM Original 0.005: CHASM qvalue of 0.005 used as cutoff to classify the datasets
CHASM Original 0.01: CHASM qvalue of 0.01 used as cutoff to classify the datasets
CHASM Original 0.05: CHASM qvalue of 0.05 used as cutoff to classify the datasets

Table 2
Composition of the datasets used as proxies to compare the performance of transformed and original scores at assessing the functional impact of cancer somatic mutations.

Name	Source	Positives	Negatives	N positives	N negatives
**WG2+/1**	Pooled cancer somatic mutations	Mutations that appear in 2 or more samples	Mutations that appear in 1 sample	1031	26025
**WGCGC/ nonCGC**	Pooled cancer somatic mutations	Mutations in genes included in the Cancer Gene Census [13]	Non-recurrent mutations in genes not included in the Cancer Gene Census	1412	24837
**WG2+/1***	Pooled cancer somatic mutations after removal of mutations within the training set of CHASM	Mutations that appear in 2 or more samples	Mutations that appear in 1 sample	946	25296
**WGCGC/ nonCGC***	Pooled cancer somatic mutations after removal of mutations within the training set of CHASM	Mutations in genes included in the Cancer Gene Census [13]	Non-recurrent mutations in genes not included in the Cancer Gene Census	1234	24834

WG: (Whole genome) Dataset of somatic mutations pooled from different tumor exome-sequencing projects (see Table 1 in main text)
