# Supplementary material for: Improving the prediction of the functional impact of cancer mutations by baseline tolerance transformation
Source: Genome Med. 2012 Nov 26;4(11):89. doi: 10.1186/gm390 (PMC4064314; doi:10.1186/gm390)
Supplement: Additional file 8 — A table showing the Matthew's correlation coefficients and overall accuracy of transformed FISs on a dataset of disease-related nsSNVs and polymorphisms. [file gm390-S8.PDF]

Additional File 8

Performance exhibited by the three transformed scores (transFIC) in the classification of the dataset of Disease/Polymorphisms nsSNVs.

	Original		GOBP		GOMF		CP		Doms	
	**MCC**	**ACC**	**MCC**	**ACC**	**MCC**	**ACC**	**MCC**	**ACC**	**MCC**	**ACC**
**SIFT**	0.42	0.638	0.416	0.703	0.409	0.7	0.41	0.695	0.414	0.702
**PPH2**	0.497	0.74	0.494	0.743	0.49	0.742	0.505	0.747	0.488	0.734
**MA**	0.548	0.786	0.523	0.767	0.488	0.759	0.519	0.774	0.488	0.758
