# Supplementary material for: Improving the prediction of the functional impact of cancer mutations by baseline tolerance transformation
Source: Genome Med. 2012 Nov 26;4(11):89. doi: 10.1186/gm390 (PMC4064314; doi:10.1186/gm390)
Supplement: Additional file 9 — Two tables showing the values of sensitivity and specificity attained by the transFIC of the three methods when separating highly recurrent from non-recurrent COSMIC mutations and recurrent from non-recurrent COSMIC mutations. [file gm390-S9.PDF]

Additional File 9

Sensitivity and specificity attained by the transFIC of the three methods when separating Cosmic highly-recurrent from Cosmic non-recurrent, and Cosmic recurrent from Cosmic non-recurrent mutations (from Figure 4 of the main paper).

Cosmic5+/1						
	L/M			M/H		
Method	Cutoff	Sensitivity	Specificity	Cutoff	Sensitivity	Specificity
SIFT	-1	0.961	0.097	2	0.582	0.761
PPH2	-1	0.962	0.178	1.5	0.513	0.76
MA	-1	0.967	0.147	2	0.863	0.784

Cosmic2+/1						
	L/M			M/H		
Method	Cutoff	Sensitivity	Specificity	Cutoff	Sensitivity	Specificity
SIFT	-1	0.926	0.097	2	0.422	0.761
PPH2	-1	0.907	0.178	1.5	0.406	0.76
MA	-1	0.949	0.147	2	0.711	0.784

L/M: Measured at the Low impact/Medium impact border
M/H: Measured at the Medium impact/High impact border
